# A Longitudinal Study on Loneliness during the COVID-19 Pandemic in Japan

**DOI:** 10.3390/ijerph191811248

**Published:** 2022-09-07

**Authors:** Sumeet Lal, Trinh Xuan Thi Nguyen, Abdul-Salam Sulemana, Pattaphol Yuktadatta, Mostafa Saidur Rahim Khan, Yoshihiko Kadoya

**Affiliations:** School of Economics, Hiroshima University, 1-2-1 Kagamiyama, Higashihiroshima 7398525, Japan

**Keywords:** loneliness, socioeconomic factors, COVID-19 pandemic, longitudinal study

## Abstract

The prolonged COVID-19 pandemic has exacerbated existing socioeconomic and health risk factors and added additional dimensions to the loneliness problem. Considering the temporal extension of COVID-19, which exposes people to various loneliness conditions, we examined the development of loneliness and changing risk factors based on age and gender. We used longitudinal data from Hiroshima University’s nationwide survey in Japan, conducted before and during the pandemic, to categorize loneliness into three types: long-term (feeling of loneliness experienced both before and during the pandemic), post-pandemic (feeling of loneliness experienced throughout the whole pandemic period), and fresh (feeling of loneliness experienced only in the last year of the pandemic). Loneliness categorization is important because the prolonged existence of the COVID-19 pandemic has added additional dimensions to the loneliness problem, which existing studies rarely identify. As a result, the distinction between long-term and fresh loneliness remains unexplained. The weighted logit regression results revealed that many Japanese people have remained or became lonely during the pandemic and identified variations based on gender, age, and changes in socioeconomic and health characteristics. More precisely, almost 52% of the participants experienced long-term loneliness, while 8% of the participants experienced post-pandemic loneliness, and nearly 5% experienced fresh loneliness. Age and having children were associated with long-term loneliness; gender, age, leaving full-time employment, financial literacy, change in health status, and change in depression were associated with post-pandemic loneliness; and gender, having children, living in rural areas, change in household assets, financial literacy, changes in health status, and changes in depression were associated with fresh loneliness. These results indicated that long-term, post-pandemic, and fresh loneliness have distinct characteristics. The Japanese government should devise distinctive solutions for people suffering from varying loneliness before and during the pandemic rather than adopting a generalized approach.

## 1. Introduction

Existing studies on loneliness during the COVID-19 pandemic have rarely distinguished between long-term and new loneliness (post-pandemic and fresh) caused by the pandemic. Long-term loneliness refers to the feeling of loneliness both before and during the pandemic, while post-pandemic loneliness refers to the feeling of loneliness during the entire period of the pandemic, and fresh loneliness refers to the feeling of loneliness during the last year of the pandemic. This distinction is necessary, as the prolonged COVID-19 pandemic has added additional dimensions to the loneliness problem [1]. For example, changes in government’s restrictive measures, perceived risk factors, and socio-economic conditions during the entire pandemic period exhibit different consequences for the post-pandemic and fresh loneliness. New dimensions of risk factors emerged due to the strict government restrictions and peoples’ higher level of anxiety about the disease exposing them to psychological repercussions such as loneliness. However, in the last year of the pandemic, the government’s restrictive measures and peoples’ anxiety about the disease were greatly reduced due to the successful vaccination program, but changes in socio-economic conditions and psychological status posed a different level of risk factor for loneliness.

Loneliness refers to not only the state of being lonely but also the feeling of deprivation of social connectedness, which the changing socioeconomic scenarios during the pandemic are likely to affect. Loneliness has long been a public health concern [2,3] with several known risk factors, such as age, gender, lower education levels, inadequate financial resources, and limited social contact or network type [4,5,6]. The implementation of health safety measures at varying magnitudes during different phases of the pandemic, along with changes in living conditions, earning capacity, employment status, anxiety, depression, and other factors, have affected people’s psychological conditions, making them more prone to loneliness [7,8]. Several studies conducted during the COVID-19 pandemic provided evidence of increased loneliness and new risk factors such as being single, being a student, living alone, having few close friends, having no children, and residing in urban areas [9,10,11].

Most recently, using pre- and post-pandemic datasets, Khan et al. [1] noted significant differences in loneliness across age and gender subsamples and found that younger people generally had greater loneliness, whereas older people became lonelier during the pandemic in Japan. However, there is a lack of comprehensive longitudinal studies that identify factors associated with long-term loneliness and compare them with post-pandemic and fresh loneliness. Lampraki et al. [12] suggested that a longitudinal analysis during the prolonged phase of COVID-19 would effectively capture dynamics in social structures and population characteristics, which would serve as an important backdrop for determination of different conditions loneliness. Therefore, to fill this gap, this study classified loneliness into three categories: long-term, post-pandemic, and fresh loneliness. We performed a longitudinal comparison of the influence of various changes in socioeconomic conditions on different loneliness conditions.

Since the beginning of the pandemic, loneliness has become an important issue due to social distancing measures and restrictions on physical movement. Several studies have investigated the magnitude of loneliness during the pandemic compared with the pre-pandemic situation. These studies can be used as a basis to examine how new loneliness conditions are formed during the prolonged phase of COVID-19 and which groups of people are exposed to it. A study with 6000 participants in Germany, conducted two years prior to the pandemic and one year after the onset of the pandemic, revealed that compared to pre-pandemic levels, loneliness rose dramatically but began to fall before social distancing was relaxed [13]. Moreover, women, younger people, outgoing people, anxious people, and conscientious people experienced greater increases in loneliness [13]. A longitudinal study in the USA compared 189 observations collected in June 2019, before COVID-19, with data collected in June 2020, during COVID-19, and revealed an overall increase in loneliness during the COVID-19 pandemic, with certain groups of individuals with particular social network characteristics experiencing smaller increases in loneliness [14]. More specifically, people with fewer than five “extremely close” relationships expressed rising levels of loneliness, and smaller increases in loneliness during the pandemic were associated with face-to-face encounters and the length and frequency of interactions with extremely close people [14]. Conversely, a study involving 31,064 participants in the UK indicated that, before and throughout the pandemic, the risk factors for loneliness were essentially the same. Risk factors for higher loneliness were observed among young adults, students, women, people with a lower education level or income, economically inactive people, people living alone, and people living in urban areas. People who were already at risk of becoming lonely (people aged 18–30 years, people with lower household income, and adults living alone) experienced aggravated loneliness conditions during the pandemic [9]. Overall, these studies suggest that in addition to suffering from loneliness before the pandemic, many individuals continue to experience loneliness throughout the prolonged phase of the pandemic (long-term loneliness). Many people who were not lonely before the pandemic became lonely due to changing individual characteristics, leading to the development of different conditions of loneliness.

Japan has passed through various phases of the pandemic and has experienced different levels of restrictions on movement and other socioeconomic affairs [15]. Loneliness has been a long-term public health concern for Japan, particularly among the younger population [16]. A high level of loneliness persists in Japan due to changes in age- and gender-specific socioeconomic and health characteristics caused by COVID-19 in its prolonged phase [1,17]. This is compounded by the fact that Japan is a collectivist and health-conscious society, and the further intensification of loneliness is the price of complying with COVID-19 safety measures, which are necessary to keep people and society safe [18]. A recent government survey demonstrated that over 35% of people in Japan feel lonely and isolated due to the prolonged COVID-19 pandemic, and young people in their 20s and 30s have experienced a higher degree of loneliness than older people due to limited social contact [19]. Lower income, unemployment, living alone, death of a family member, feeling sick, and going to a new school or job have also been cited among the reasons. Over 20% of people also reported that their mental health worsened last year due to the pandemic [19]. As the prolonged COVID-19 pandemic continues to expose people to loneliness, it is of paramount importance to analyze the development of different loneliness conditions and the influence of changing risk factors based on age and gender on loneliness.

This study contributes to the existing body of literature in at least two ways. First, to the best of our knowledge, this is the first study that categorizes loneliness into three groups depending on the phases of the COVID-19 pandemic and provides detailed longitudinal evidence on how men and women of different age groups are exposed to these loneliness conditions. Second, this study reveals that socioeconomic and health risk factors have varying associations with various loneliness conditions, particularly across broader age and gender subsamples.

## 2. Theoretical Background

Loneliness is a persistent public health problem that not only negatively impacted individual mental and physical health but also deteriorated social cohesion and public trust. In light of the prolonged COVID-19 pandemic and the development of different loneliness conditions, a thorough understanding of the phenomena and its primary causes is necessary to adequately address the complex and multidimensional problem of loneliness. Several studies observed loneliness from the viewpoint of social dynamics. For example, Beutel et al. [20] argued that age, gender, not having children or a partner, living conditions, socio-economic status, smoking, and psychological distress are major determinants of loneliness in Germany. Franssen et al. [21] found that living alone, a lower frequency of contact with neighbors, social exclusion, psychological distress, lower emotional and psychological well-being, employment status, and marital status influenced loneliness in the Netherlands. During the COVID-era, a longitudinal study conducted in the UK from 2017–2020 revealed that loneliness is higher during the COVID-19 pandemic, but the risk factors are nearly identical to before the pandemic. Age and income were negatively associated with loneliness both before and during the pandemic, while living alone, being female, lower education, and living in urban areas were positively associated with loneliness both before and during the pandemic, and all these effects were strengthened during the pandemic. Furthermore, being unemployed, inactive, or students were factors associated with loneliness before and during the pandemic [9]. Findings of all these studies indicate that potential risk factors of loneliness could be impacted by the prolonged COVID-19 pandemic in Japan, causing people to experience different levels and conditions of loneliness overtime.

The implementation of health safety measures, such as social distancing and lockdown, plays an important role in influencing loneliness. However, the direction of loneliness varies by country and level of execution. For example, a study conducted in the USA in January 2020, before the pandemic began, and in late March and April revealed that there were no significant mean-level changes in loneliness in all three assessments and that older adults reported less overall loneliness compared to younger age groups but had an increase in loneliness during the acute phase of the outbreak [22]. Their loneliness, however, leveled off after the issuance of stay-at-home orders. Individuals living alone and those with at least one chronic condition reported feeling lonelier at the baseline but did not experience increase in loneliness during the implementation of social distancing measures [22]. Ernst et al. [23] explored whether changes such as lockdowns, physical distancing, and the switch to remote work and school during the pandemic increased people’s loneliness and found that while these measures increased social isolation, they do not always lead to loneliness. On the contrary, Hwang et al. [24] found that social distancing measures not only severely increased the levels of loneliness of older adults but also amplified their preexisting mental and physical illnesses.

As COVID-19 health safety measures have a contrasting impact on loneliness levels, policy makers may incur some political costs as they would suffer from a substantial cost-benefit trade-off while implementing these measures. This is because these measures not only influence loneliness levels but also might prove ineffective in curtailing COVID cases or deaths, which could lead to erosion of public trust. Allen [25] examined over 100 COVID-19 studies and found that many relied on false assumptions that over-estimated the benefits and under-estimated the costs of lockdown. His study concludes that lockdown is not effective in limiting COVID-19 deaths and that it adds more costs, such as higher levels of loneliness, consequently classifying lockdown as one of the greatest peacetime policy failures in modern history. In contrast, Gandjour [26] determined the clinical and economic value of a business shutdown that is successful in “flattening” or “squashing the COVID-19 curve” in Germany. He found that a successful shutdown yielded a considerable gain in life years in the German population and reduced mortality and COVID cases in general. The varying effects of health safety measures across countries may put decision makers in a dilemma, as their policy may warrant higher levels of loneliness or might erode public trust. Daumann et al. [27] recommended framework for political decision making under incomplete information and uncertainty and suggested that health policy that aims to provide comprehensive protection against infection should also be based on economic criteria. Japan has experienced a total of seven COVID waves in total, and the level of social distancing measures and lockdowns varied to some extent in each of the waves. Not only that, but each of these waves and levels of COVID-19 safety measures changed the socio-economic and psychological characteristics of individuals overtime, consequently exposing them to different loneliness conditions. However, the question now remains whether the benefit of all these safety measures has outweighed the associated costs such as loneliness.

## 3. Data and Methods

### 3.1. Data

This study used the Hiroshima University Household Behavioral and Financial Survey. Nikkei Research, one of Japan’s leading research firms, collected the online panel dataset. Each prospective participant received a questionnaire. The number of participants was calculated using random sampling to maintain the representativeness of the panel data. The survey asked about the preferences and demographic, socioeconomic, and psychological aspects of Japanese adults aged 20 years or older before and during the COVID-19 pandemic (2020, 2021, and 2022). The sample sizes in the three waves were 17,463, 6103, and 4281 participants, respectively. We merged the three datasets; after removing socioeconomic missing values of financial literacy, household income, household assets, and employment status, the final number of observations was 2630.

### 3.2. Variables

To examine how socioeconomic factors affected loneliness over the surveyed period, we created three dependent variables: long-term loneliness, indicating loneliness during all 3 years; post-pandemic loneliness, indicating lack of loneliness before the pandemic in March 2020, followed by loneliness in 2021 and 2022; and fresh loneliness, indicating lack of loneliness in the first two years, followed by loneliness in 2022.

This study used explanatory variables similar to those included in previous studies [1,17]. Demographic variables, such as gender, child-rearing status, living regions, and educational attainment, were extracted from the 2020 wave, whereas socioeconomic variables, such as age, marital status, living alone, employment, household income, and household assets, were obtained from the 2020 and 2022 datasets. Furthermore, as financial literacy was reported to be a proxy for rational financial and health behaviors [28,29,30], the variable of financial literacy from the 2020 wave was included in our study. The remaining variables were related to the subjective evaluation of financial and health status, including the subjective evaluation of health, anxiety about the future, financial satisfaction, and a myopic view of the future. Table 1 provides a detailed description of each variable.

### 3.3. Descriptive Statistics

The descriptive statistics shown in Table 2 indicated that 52% of the participants experienced long-term loneliness. Furthermore, 8% of the participants experienced post-pandemic loneliness, and nearly 5% experienced fresh loneliness. Nearly 70% of the participants were men, with an average age of 54 years, with a 15-year education on average, achieving a financial literacy score of 0.7, and rating scores for their perception of the future of 2.69 out of 5. Approximately 57.3% of the participants lived in rural areas, and approximately 3% left full-time jobs. Regarding household composition, 1.3% left their spouse, 1.9% started living alone, and 59% had children. Regarding household financial status, most participants had households with a total asset value of JPY 24.1 million and a total annual income of JPY 6.50 million. Regarding changes in subjective assessments of financial and health status, 25.6% of the participants rated their health conditions poorer during the pandemic, 30% became more anxious about the future, and 29.2% experienced worsening depression from 2020 to 2022.

The entire sample was separated into subsamples stratified by gender and age. Table 3, Table 4 and Table 5 describe the distribution of long-term, pandemic-related, and fresh loneliness, respectively. Long-term loneliness varied significantly across gender and age groups. In particular, the percentage of people under 65 years of age who experienced long-term loneliness was higher than that of older people irrespective of gender. However, there was no statistically significant difference between the younger and older subsamples with post-pandemic loneliness. Nevertheless, a disparity at the 90% significance level was observed among people with fresh loneliness, with a higher proportion of older than younger individuals.

### 3.4. Methods

We adopted the following equations to analyze the association between different types of loneliness and participants’ demographic, socioeconomic, psychological, and health-related factors:

Y1i = f(Xi, ∆Xi, εi)(1)

Y2i = f(Xi, ∆Xi, εi)(2)

Y3i = f(Xi, ∆Xi, εi)(3)

where Y1i is a measure of long-term loneliness in 2020, 2021, and 2022; Y2i is a measure of post-pandemic loneliness; Y3i is a measure of fresh loneliness; X is a vector of individuals’ demographic, socioeconomic, psychological, and health-related characteristics; ∆X is a vector of change in individuals’ demographic, socioeconomic, psychological, and health-related characteristics from 2020 to 2022; and ε is the error term. We tracked the transformations of different loneliness conditions across the surveyed period of three years using weighted logit regression models, as all dependent variables were binary. This study used weighted logit regression to ensure the adequate representation of each group, with sampling weights calculated as a fraction of the total population divided by age group and gender. The total population in Japan in 2021 was extracted from the latest dataset, based on the 2020 Population Census [31]. Weighted logit regression was performed by combining the sampling weights into the logit regression models.

To avoid any potential intercorrelation issues among the independent variables, we conducted correlation and multicollinearity tests for all models (results available upon request). Our findings showed weak correlation between the explanatory variables (<0.7) without multicollinearity in all models (variance inflation factor < 3).

We did not use the changed form of the myopic view of the future, as future orientation did not significantly change over time. Specifically, we employed a myopic view of the future in 2020, 2021, and 2022 for the regression analysis of long-term, post-pandemic, and fresh loneliness, respectively. The full specifications for Equations (1)–(3) are represented in Models (4)–(6), respectively.

Long-term lonelinessi20,21,22 = β0 + β1malei + β2agei + β3recently divorcedi + β4childreni + β5becoming alonei + β6living in rural areasi + β7educationi + β8left full-time emplymenti + β9logofchange inhousehold incomei + β10logofchange in household assetsi + β11financial literacyi + β12change in health statusi + β13change in future anxietyi + β14change in financial satisfactioni + β15change in depressioni + β16myopic view of the futurei + εi(4)

Post-pandemic lonelinessi21,22 = β0 + β1malei + β2agei + β3recently divorcedi + β4childreni + β5becoming alonei + β6living in rural areasi + β7educationi + β8left full-time emplymenti + β9logofchange inhousehold incomei + β10logofchange in household assetsi + β11financial literacyi + β12change in health statusi + β13change in future anxietyi + β14change in financial satisfactioni + β15change in depressioni + β16myopic view of the futurei + εi(5)

Fresh lonelinessi22 = β0 + β1malei + β2agei + β3recently divorcedi + β4childreni + β5becoming alonei + β6living in rural areasi + β7educationi + β8left full-time emplymenti + β9logofchange inhousehold incomei + β10logofchange in household assetsi + β11financial literacyi + β12change in health statusi + β13change in future anxietyi + β14change in financial satisfactioni + β15change in depressioni + β16myopic view of the futurei + εi(6)

## 4. Results

To gain a greater understanding of the changing effects of various socioeconomic, demographic, psychological, and health-related factors on different loneliness conditions, we conducted a panel data regression analysis for the three dependent variables: long-term, post-pandemic, and fresh loneliness. The results are presented in Table 6. Data with missing values for recent divorce and starting to live alone were excluded from the regression analysis of post-pandemic and fresh loneliness, respectively.

Overall, we found that being male was the only variable that had a positive association with post-pandemic loneliness, whereas the negative association with age was relatively consistent across the three types of loneliness. Moreover, a statistically significant and negative relationship was observed between financial literacy and post-pandemic loneliness, indicating that people with higher levels of financial literacy tended to be less lonely due to the impact of the pandemic.

In addition, we conducted subsample analyses based on gender and age, as shown in Table 7 and Table 8, respectively, to investigate the effects of gender and age on the association between different loneliness conditions and various socioeconomic, demographic, psychological, and health-related factors. The findings demonstrated that there was heterogeneity in the signs and significance of the association between variables and three types of loneliness across age and gender.

Men had a highly significant and positive association with long-term loneliness and post-pandemic loneliness in elderly people. Having children was negatively associated with long-term loneliness regardless of gender and age. Leaving full-time jobs was negatively associated only with post-pandemic loneliness. However, the sign of the relationship between various loneliness conditions and getting divorced, living in rural regions, household income, and worsening depression fluctuated widely across subsamples. Moreover, while lower financial satisfaction and a myopic view of the future were positively associated with post-pandemic and long-term loneliness, respectively, a negative association was observed between worsening health status and post-pandemic loneliness only among women and young people.

Robust standard errors (SE) of the coefficients of each independent variable are provided in parentheses of Table 6, Table 7 and Table 8. We used robust standard errors because it solves the heteroskedasticity issue and provides a more accurate measure of standard errors. The low robust standard errors, particularly for the significant variables, indicate that the sample used in our study is reliable and reflects the population well.

## 5. Discussion

### 5.1. Common Covariates of Loneliness Conditions

This study used data from a longitudinal survey collected annually between 2020 and 2022 to examine the prevalence of different types of loneliness during the COVID-19 pandemic and its association with demographic, socioeconomic, and other psychological factors. The results demonstrated that loneliness arising from the impact of the COVID-19 pandemic in Japan requires more public recognition. Nearly half of loneliness cases were categorized as long-term, and a significant number were starting to be lonely.

Furthermore, the results indicated that being male and having higher financial literacy showed a significant correlation with most types of loneliness. We observed a positive correlation between being male and all three types of loneliness. The positive correlation between long-term loneliness and older men was in line with an earlier study that reported that older men had higher levels of loneliness before the pandemic and continued to experience a slight increase in loneliness over time [32]. The same held true for post-pandemic and fresh loneliness, with the majority of affected individuals being young men. Okun and Keith [33] suggested that women in general were more likely to have larger and more active social networks, which consequently protected them from loneliness in times of crisis. Barreto et al. [34] argued that young men who were individualistic in nature experienced the highest levels of loneliness. Hence, amidst the prolonged COVID-19 pandemic, it is understandable why Japanese men, particularly young men with strong individualistic values, experienced greater loneliness [35]. We found that financial literacy was negatively associated with post-pandemic and fresh loneliness, particularly among young people. Young generations with higher financial literacy were unaffected by long-lasting financial strain during the COVID-19 pandemic, which protected them from becoming lonely [36,37]. Hence, financial literacy, as a tool for rational decision making, prevented young men from financial strain, promoted cognitive social capital and optimism, and suppressed loneliness. The same did not hold true for men over 65 years of age, as literacy or knowledge diminishes with age; thus, these older individuals might possibly suffer from financial strain, which could ultimately contribute to resource loneliness.

Several other variables showed inconsistent relationships with loneliness. Our study revealed that older people with a higher household income were less likely to have long-term loneliness, which was consistent with studies that found that socioeconomically privileged people were at a lower risk of being lonely [38,39]. However, a similar pattern was not observed for people with post-pandemic loneliness, which could be attributed to the fact that people with higher incomes and contentment with their finances may have seen variations in their social lifestyles due to the pandemic and may have adjusted quickly with time [17]. Worsening health status and higher levels of depression appear to have varied relationships depending on loneliness type. It is unclear what accounts for this difference although this finding was in line with an earlier study that showed divergent associations between changes in subjective health assessment and depression and loneliness, mediated by the effects of age and gender [1].

### 5.2. Specific Covariates of Loneliness Conditions

We discovered significant associations between long-term loneliness, having children, and myopic views of the future. People living with their children were less likely to suffer from chronic loneliness. It is evident that people with children continued to have social interactions, which were not interrupted by the pandemic; therefore, they likely felt companionship, which prevented them from developing continued loneliness. This correlation has also been demonstrated in other studies, which showed that a higher contact frequency with children or grandchildren reduced loneliness [12,40]. Furthermore, we found that myopic views of the future were significantly positively correlated with long-term loneliness among men. As men are not as futuristic as women [41], they were in vulnerable positions and more exposed to long-term loneliness. Hence, a shift away from a myopic view to future thinking can make an individual more resilient and better prepared for unforeseen crisis management [42]. This can be achieved by developing a sense of understanding that current living is not all, but the future has many benefits as well. Educating children in schools about the benefits of futuristic thinking and activities could generate a sense of long-term vision in them.

In terms of post-pandemic loneliness, our findings showed significant results for exiting full-time jobs and changes in financial satisfaction. First, surprisingly, we found that people who quit full-time employment were less likely to experience loneliness. One explanation is that considering the prominent effects of changing working patterns, especially working from home as a freelancer, and fear of losing family members during the COVID-19 pandemic, the decision to leave full-time jobs and start temporary positions or stay unemployed could be justifiable. Furthermore, most studies reporting the positive relationship between quitting full-time jobs and loneliness were cross-sectional [43]; therefore, a longitudinal study exploring the complex link between loneliness and employment status is necessary in the future. Second, Wang et al. [44] demonstrated that individuals showing negative emotions, such as anxiety and disappointment, were likely to suffer from loneliness. This was in line with our finding that younger generations who experience lower levels of financial satisfaction were more susceptible to loneliness, as the young are easily vulnerable to immediate societal changes during the pandemic, ranging from school closures to job conditions.

Moreover, our results indicated significant associations between fresh loneliness, getting divorced, and living in rural areas. Regarding getting divorced, the positive association was found particularly among elderly people, which corresponded to other studies that reported that people without a spouse or co-habiting partner had greater loneliness [20,45,46,47]. However, our results showed that younger people who lived in rural areas and were not lonely in the first two years had a lower likelihood of developing loneliness in 2022. This might contradict a study that emphasized the need to address loneliness issues for underprivileged people in rural areas [48]. However, it is possible that the relationship with loneliness among rural inhabitants was mediated by social technology and the frequency of social contact [49], which means that with the high coverage of social media use and the less-severe COVID-19 situation, young Japanese people from rural regions can easily afford the opportunity to cultivate social connection compared to their urban counterparts.

This study had several limitations. First, self-reported questionnaires were used to identify loneliness, depression, and anxiety instead of an outlined clinical test, which is an essential criterion in the psychiatric diagnosis scale. However, the reliability and discriminatory validity of the questionnaire have been justified in previous studies [1,17]. Second, our data were collected from a web-based survey; therefore, we cannot dismiss the possibility of limited participation, especially among those with socioeconomic disadvantages. Third, some subsamples had low observation numbers, such as a much greater proportion of men than women. Although we ran a weighted data analysis to reduce the effect of underrepresented groups, the associations of the three types of loneliness and socioeconomic factors in this study could be conservative to a certain extent.

## 6. Conclusions

In the context of the prolonged existence of the COVID-19 pandemic, we investigated various levels of loneliness and their covariates in Japan. This study showed that a significant proportion of Japanese people remained lonely or became lonely during the COVID-19 pandemic and that different socioeconomic characteristics have different effects on different loneliness conditions. More specifically, age and having children were associated with long-term loneliness; gender, age, leaving full-time employment, financial literacy, change in health status, and change in depression were associated with post-pandemic loneliness; and gender, having children, living in rural areas, change in household assets, financial literacy, changes in health status, and changes in depression were associated with fresh loneliness. These results highlight the necessity of a more intensive focus on loneliness as a public health concern in Japan. Recent interventional programs, such as promoting consultation services to lonely people and their families, are underway to mitigate the growing problem of loneliness and are a priority in Japan [50]. However, as long-term, post-pandemic, and fresh loneliness have unique characteristics, which should be explored from the perspective of the associated variables, our study suggests that the Japanese government should devise catered solutions for people who suffer from loneliness, either before or during the pandemic, rather than using a generalized approach. This can be achieved through low-tech community-based programs, high-tech digital approaches, and nurse-led care coordination models. Future studies are required to provide evidence on interventions targeting groups of people who experience distinct loneliness conditions.

## Figures and Tables

**Table 1:** Variable definitions.

Variables	Definition
	Dependent variables
Long-term loneliness	Binary variable: 1 = feeling lonely in all three years (2020, 2021, 2022), and 0 = otherwise
Post-pandemic loneliness	Binary variable: 1 = not feeling lonely in 2020 but becoming lonely in 2021 and remaining in that condition in 2022, and 0 = otherwise
Fresh loneliness	Binary variable: 1 = not feeling lonely in 2020 and 2021 but becoming lonely in 2022, and 0 = otherwise
	Explanatory variables
Male *	Binary variable: 1 = male and 0 = female
Age *	Continuous variable: participants’ age in 2022
Recently divorced	Binary variable: 1 = divorced in 2022, and 0 = otherwise
Children *	Binary variable: 1 = at least one child, and 0 = otherwise
Started living alone	Binary variable: 1 = recently started living alone in 2022, and 0 = otherwise
Living in rural areas *	Binary variable: 1 = live in a rural area (not in Tokyo special wards or government-designated city areas), and 0 = otherwise
Education *	Discrete variable: years of education
Left full-time employment	Binary variable: 1 = recently left a full-time job, and 0 = otherwise
Household income	Continuous variable: annual earned income before taxes and with bonuses of the entire household (unit: JPY)
Log of change in household income	Log (change in household income from 2020 to 2022)
Household asset	Continuous variable: balance of financial assets (savings, stocks, bonds, insurance, etc.) of the entire household (unit: JPY)
Log of change in household asset	Log (change in household asset from 2020 to 2022)
Financial literacy *	Continuous variable: average scores of answers for the three financial literacy questions
Subjective health status	Ordinal variable for the statement “I am now healthy and was generally healthy in the last one year”; responses: 1 = not true at all, 2 = not so true, 3 = neutral, 4 = somewhat true, and 5 = true
Change in health status	Binary variable: 1 = experiencing worsening health conditions, and 0 = otherwise
Future anxiety	Ordinal variable for the statement “I have anxieties about ‘life after 65 years of age’ (for those who were already aged 65 years or above, ‘life in the future’)”; responses: 1 = not true at all, 2 = not so true, 3 = neutral, 4 = somewhat true, and 5 = true
Change in future anxiety	Binary variable: 1 = becoming more anxious about the future, and 0 = otherwise
Financial satisfaction	Ordinal variable for the statement “I am happy with my financial status”; responses: 1 = completely disagree, 2 = disagree, 3 = neutral, 4 = agree, and 5 = completely agree
Change in financial satisfaction	Binary variable: 1 = having lower financial satisfaction levels, and 0 = otherwise
Depression	Ordinal variable for the statement “I often feel depressed or felt depressed in the last year”; responses: 1 = not true at all, 2 = not so true, 3 = neutral, 4 = somewhat true, and 5 = true
Change in depression	Binary variable: 1 = having worsening depression, and 0 = otherwise
Myopic view of the future	Ordinal variable for the statement “Since the future is uncertain, it is a waste to think about it”; responses: 1 = completely disagree, 2 = disagree, 3 = neutral, 4 = agree, and 5 = completely agree

Note: * indicates data from the 2020 wave.

**Table 2:** Descriptive statistics.

Variables	Mean	Std. Dev.	Min	Max
Dependent variables				
Long-term loneliness	0.5213	0.4996	0	1
Post-pandemic loneliness	0.0852	0.2792	0	1
Fresh loneliness	0.0475	0.2128	0	1
Explanatory variables				
Male	0.6970	0.4597	0	1
Age	53.8266	12.7165	22	87
Recently divorced	0.0133	0.1146	0	1
Children	0.5916	0.4916	0	1
Started living alone	0.0194	0.1379	0	1
Living in rural areas	0.5726	0.4948	0	1
Education	15.0177	2.0961	9	21
Left full-time employment	0.0338	0.1809	0	1
Household income	6,511,217	4,262,293	500,000	21,000,000
Household asset	24,100,000	31,900,000	1,250,000	125,000,000
Financial literacy	0.7099	0.3305	0	1
Change in health status	0.2563	0.4367	0	1
Change in future anxiety	0.3000	0.4583	0	1
Change in financial satisfaction	0.2129	0.4095	0	1
Change in depression	0.2928	0.4551	0	1
Myopic view of the future	2.6882	1.0048	1	5
Observations	2630			

**Table 3:** Distribution of long-term loneliness by gender and age group.

Long-Term Loneliness	Male		Female		Total
	Younger (<65)	Older (≥65)	Younger (<65)	Older (≥65)	
0	588	297	301	73	1259
	43.88%	60.24%	43.31%	71.57%	47.87%
1	752	196	394	29	1371
	56.12%	39.76%	56.69%	28.43%	52.13%
Total	1340	493	695	102	2630
	100%	100%	100%	100%	100%
Mean difference	t = 6.2796 ***		t = 5.4318 ***		
	F = 23.07 ***				

Note: *** *p* < 0.01.

**Table 4:** Distribution of post-pandemic loneliness by gender and age group.

Post-Pandemic Loneliness	Male		Female		Total
	Younger (<65)	Older (≥65)	Younger (<65)	Older (≥65)	
0	1211	452	647	96	2406
	90.37%	91.68%	93.09%	94.12%	91.48%
1	129	41	48	6	224
	9.63%	8.32%	6.91%	5.88%	8.52%
Total	1340	493	695	102	2630
	100%	100%	100%	100%	100%
Mean difference	t = 0.8573		t = 0.3839		
	F = 1.79				

**Table 5:** Distribution of fresh loneliness by gender and age group.

Fresh Loneliness	Male		Female		Total
	Younger (<65)	Older (≥65)	Younger (<65)	Older (≥65)	
0	1286	458	667	94	2505
	95.97%	92.90%	95.97%	92.16%	95.25%
1	54	35	28	8	125
	4.03%	7.10%	4.03%	7.84%	4.75%
Total	1340	493	695	102	2630
	100%	100%	100%	100%	100%
Mean difference	t = −2.7152 ***		t = −1.7333 *		
	F = 3.51 *				

Note: *** *p* < 0.01, * *p* < 0.1.

**Table 6:** Regression results for long-term, post-pandemic, and fresh loneliness.

Explanatory Variables	Dependent Variables		
	Long-Term Loneliness	Post-Pandemic Loneliness	Fresh Loneliness
Male	0.0605	0.543 **	0.749 **
	(0.161)	(0.238)	(0.365)
Age	−0.0203 ***	−0.0178 **	−0.00588
	(0.00604)	(0.00763)	(0.0108)
Recently divorced	−0.0986	−	1.385
	(0.469)		(0.859)
Children	−0.444 ***	−0.123	0.382 *
	(0.114)	(0.175)	(0.232)
Started living alone	0.132	−1.125	−
	(0.452)	(0.798)	
Living in rural areas	−0.00913	0.335	−0.666 **
	(0.149)	(0.216)	(0.267)
Education	0.0473	0.0242	−0.0894
	(0.0397)	(0.0456)	(0.0867)
Left full-time employment	0.0216	−1.567 ***	0.168
	(0.270)	(0.598)	(0.571)
Log of change in HH income	−0.0852	0.0647	−0.165
	(0.174)	(0.234)	(0.170)
Log of change in HH assets	−0.00672	−0.111	0.324 *
	(0.111)	(0.155)	(0.193)
Financial literacy	0.169	−0.762 **	−0.833 **
	(0.206)	(0.343)	(0.372)
Change in health status	0.0260	−0.747 ***	0.642 *
	(0.172)	(0.266)	(0.337)
Change in future anxiety	−0.0769	−0.0268	−0.0925
	(0.151)	(0.212)	(0.266)
Change in financial satisfaction	0.273	0.426	−0.526
	(0.196)	(0.281)	(0.376)
Change in depression	0.0668	−0.743 ***	−0.781 **
	(0.160)	(0.230)	(0.316)
Myopic view of the future	0.0697	−0.0816	−0.0453
	(0.0684)	(0.0921)	(0.118)
Constant	0.0563	−1.464 **	−0.807
	(0.783)	(0.698)	(1.752)
Observations	2630	2595	2579
Log pseudolikelihood	−63,520,195	−25,608,303	−19,447,071
Chi^2^ statistics	53.94	38.04	33.21
*p*-value	0.0000	0.0009	0.0044

Note: Robust standard errors in parentheses. *** *p* < 0.01, ** *p* < 0.05, * *p* < 0.1.

**Table 7:** Regression results for long-term, post-pandemic, and fresh loneliness, stratified by gender.

Variables	Long-Term Loneliness		Post-Pandemic Loneliness		Fresh Loneliness	
	Male	Female	Male	Female	Male	Female
Age	−0.00803	−0.0322 ***	−0.0205 *	−0.0143	−0.0150	0.0149
	(0.00784)	(0.00860)	(0.0106)	(0.0105)	(0.0150)	(0.0130)
Recently divorced	−0.0348	−0.00502	−	−	−	2.240 **
	(0.533)	(0.646)	−	−	−	(1.013)
Children	−0.328 **	−0.595 ***	−0.0829	−0.276	0.336	0.538
	(0.129)	(0.190)	(0.219)	(0.306)	(0.257)	(0.476)
Started living alone	0.528	−0.312	−0.839	−	−	−
	(0.521)	(0.778)	(0.837)	−	−	−
Living in rural areas	−0.0436	0.00748	0.723 ***	−0.0587	−0.569 *	−0.565
	(0.177)	(0.202)	(0.280)	(0.331)	(0.304)	(0.389)
Education	0.00937	0.0560	0.0710	−0.0522	−0.124	0.0957
	(0.0409)	(0.0584)	(0.0518)	(0.0779)	(0.0854)	(0.0920)
Left full-time employment	−0.185	0.150	−0.964	−	0.720	−
	(0.315)	(0.497)	(0.631)	−	(0.653)	−
Log of change in HH income	0.196	−0.281	0.243	−0.143	−0.203	0.00171
	(0.251)	(0.224)	(0.332)	(0.237)	(0.250)	(0.285)
Log of change in HH assets	−0.0541	0.0713	−0.0762	−0.184	0.593 ***	−0.650 **
	(0.137)	(0.147)	(0.206)	(0.217)	(0.183)	(0.294)
Financial literacy	0.315	0.0889	−0.904 **	−0.622	−0.886 **	−0.771
	(0.299)	(0.266)	(0.439)	(0.513)	(0.398)	(0.674)
Change in health status	0.0172	−0.0187	−0.444	−1.172 **	0.625	0.701
	(0.227)	(0.220)	(0.307)	(0.461)	(0.422)	(0.451)
Change in future anxiety	0.161	−0.179	0.127	−0.216	−0.0398	−0.452
	(0.177)	(0.218)	(0.264)	(0.372)	(0.354)	(0.482)
Change in financial satisfaction	0.271	0.316	0.355	0.503	−0.625	−0.431
	(0.252)	(0.244)	(0.361)	(0.399)	(0.520)	(0.514)
Change in depression	0.180	−0.00953	−0.713 **	−0.794 **	−1.028 ***	−0.470
	(0.211)	(0.218)	(0.279)	(0.386)	(0.390)	(0.486)
Myopic view of the future	0.199 ***	−0.0533	−0.0937	−0.101	−0.113	0.0322
	(0.0705)	(0.124)	(0.0981)	(0.198)	(0.151)	(0.201)
Constant	−0.541	1.073	−1.810 **	−0.195	1.043	−4.824 ***
	(1.056)	(1.058)	(0.864)	(1.060)	(2.193)	(1.709)
Observations	1833	797	1810	755	1782	763
Log pseudolikelihood	−3.260 × 10^7^	−2.980 × 10^7^	−1.470 × 10^7^	−1.060 × 10^7^	−1.070 × 10^7^	−8.056 × 10^6^
Chi^2^ statistics	36.11	42.25	18.85	20.74	38.52	19.42
*p*-value	0.00170	0.000206	0.171	0.0544	0.000238	0.111

Note: Robust standard errors in parentheses. *** *p* < 0.01, ** *p* < 0.05, * *p* < 0.1.

**Table 8:** Regression results for long-term, post-pandemic, and fresh loneliness, stratified by age group.

Variables	Long-Term Loneliness		Post-Pandemic Loneliness		Fresh Loneliness	
	Younger People	Older People	Younger People	Older People	Younger People	Older People
Male	−0.136	0.801 ***	0.565 **	0.727 *	0.810 **	0.283
	(0.155)	(0.305)	(0.275)	(0.425)	(0.412)	(0.435)
Age	0.0111	−0.110 ***	−0.0188	−0.0975 **	−0.0253	−0.0256
	(0.00822)	(0.0243)	(0.0136)	(0.0465)	(0.0162)	(0.0467)
Recently divorced	0.263	−4.091 **	−	−	−	3.809 **
	(0.477)	(1.922)	−	−	−	(1.743)
Children	−0.409 ***	−0.781 **	−0.243	0.438	0.444 *	0.501
	(0.114)	(0.361)	(0.190)	(0.550)	(0.258)	(0.668)
Started living alone	0.267	2.677	−1.219	−	−	−
	(0.442)	(1.776)	(0.835)	−	−	−
Living in rural areas	0.0939	0.00761	0.439 **	0.0876	−0.631 **	−0.408
	(0.146)	(0.257)	(0.224)	(0.421)	(0.308)	(0.414)
Education	0.0116	0.00531	0.0637	−0.138	−0.149 *	0.106
	(0.0370)	(0.0667)	(0.0464)	(0.103)	(0.0872)	(0.104)
Left full-time employment	−0.0558	−0.214	−1.600 **	−1.813 *	−0.286	0.578
	(0.315)	(0.553)	(0.720)	(1.072)	(0.846)	(0.771)
Log of change in HH income	0.110	−0.684 *	−0.0814	0.877 **	−0.0694	−0.397
	(0.188)	(0.353)	(0.234)	(0.355)	(0.192)	(0.568)
Log of change in HH assets	0.0166	−0.00421	−0.0392	−0.585	0.324	0.329
	(0.109)	(0.218)	(0.164)	(0.467)	(0.207)	(0.293)
Financial literacy	0.168	0.125	−0.852 **	−0.710	−0.742 *	−0.615
	(0.215)	(0.425)	(0.340)	(0.920)	(0.397)	(0.878)
Change in health status	0.0226	0.186	−0.698 **	−0.737	0.613	0.645
	(0.171)	(0.322)	(0.296)	(0.546)	(0.376)	(0.549)
Change in future anxiety	0.0158	−0.157	−0.0433	0.0184	0.128	−0.352
	(0.142)	(0.298)	(0.220)	(0.503)	(0.321)	(0.491)
Change in financial satisfaction	0.173	0.472	0.556 *	−0.350	−0.683	0.0527
	(0.189)	(0.343)	(0.298)	(0.654)	(0.442)	(0.574)
Change in depression	0.372 **	−0.413	−0.642 **	−1.036 *	−0.973 ***	−0.394
	(0.148)	(0.301)	(0.252)	(0.579)	(0.371)	(0.533)
Myopic view of the future	0.0665	0.114	−0.0381	−0.259	−0.0599	0.0298
	(0.0714)	(0.138)	(0.0985)	(0.201)	(0.128)	(0.274)
Constant	−0.770	6.866 ***	−2.102 ***	6.441 *	0.714	−2.444
	(0.795)	(2.203)	(0.722)	(3.873)	(1.832)	(4.665)
Observations	2035	595	2006	585	1976	587
Log likelihood	−4.440 × 10^7^	−1.580 × 10^7^	−1.900 × 10^7^	−6.138 × 10^7^	−1.220 × 10^7^	−6.708 × 10^6^
Chi^2^ statistics	24.23	54.47	31.76	77.72	20.16	31.88
*p*-value	0.0846	4.34 × 10^−6^	0.00694	7.47 × 10^−11^	0.125	0.00669

Note: Robust standard errors in parentheses. *** *p* < 0.01, ** *p* < 0.05, * *p* < 0.1.

## Data Availability

Data are available upon request.
